# Supplementary material for: Isolation, Identification, and Biocontrol Potential of Root Fungal Endophytes Associated with Solanaceous Plants against Potato Late Blight (Phytophthora infestans)
Source: Plants (Basel). 2022 Jun 18;11(12):1605. doi: 10.3390/plants11121605 (PMC9229062; doi:10.3390/plants11121605)
Supplement: Supplementary file 1 [file plants-11-01605-s001.zip › plants-1775279-supplementary.pdf]

plants

Article

Isolation, Identification, and Biocontrol Potential of Root Fungal Endophytes Associated with Solanaceous Plants Against Potato Late Blight (*Phytophthora infestans*)

Abbas El-Hasan[1,*], Grace Ngatia[1], Tobias I. Link[1] and Ralf T. Voegele[1]

[1] Department of Phytopathology, Institute of Phytomedicine, Faculty of Agricultural Sciences, University of Hohenheim, Otto-Sander-Str. 5, D-70599 Stuttgart, Germany;
* Correspondence: aelhasan@uni-hohenheim.de; Tel.: +49 711 459 22392 (A.E.-H.).

Supplementary Materials

Citation: El-Hasan, A.; Ngatia, G.; Link, T.I.; Voegele, R.T. Isolation, Identification, and Biocontrol Potential of Root Fungal Endophytes Associated with Solanaceous Plants against Potato Late Blight (*Phytophthora infestans*). *Plants* **2022**, *11*, 1605. https://doi.org/10.3390/plants11121605

Academic Editors: Špela Mechora and Dragana Šunjka

Received: 1 June 2022
Accepted: 14 June 2022
Published: 18 June 2022

Publisher's Note: MDPI stays neutral with regard to jurisdictional claims in published maps and institutional affiliations.

Copyright: © 2022 by the authors. Licensee MDPI, Basel, Switzerland. This article is an open access article distributed under the terms and conditions of the Creative Commons Attribution (CC BY) license (https://creativecommons.org/licenses/by/4.0/).

Table S1. Characteristics of sampling regions.

Sampling region	Geographic coordinates	Altitude (m asl[a])	Rainfall [b] (mm)	Soil properties			Soil types
				pH	EC [c]	OM [d]	
Kilifi	3°38′S 39°51′E	8	942	7.4 – 7.6	0.02 – 0.52	0.7 – 2.0	Luvisols
Kiambu	1°14'S 36°39'E	2073	893	6.5 – 6.9	0.05 – 0.12	1.2 – 3.4	Nitisols, Andosols
Nyandarua	0°42'S 36°39'E	2540	1219	5.3 - 7.0	0.05 – 0.14	5.3 – 7.0	Cambisols, Glaysols

[a] above sea level, [b] annual average, [c] EC: electrical conductivity in µS/cm, [d] OM: organic matter.

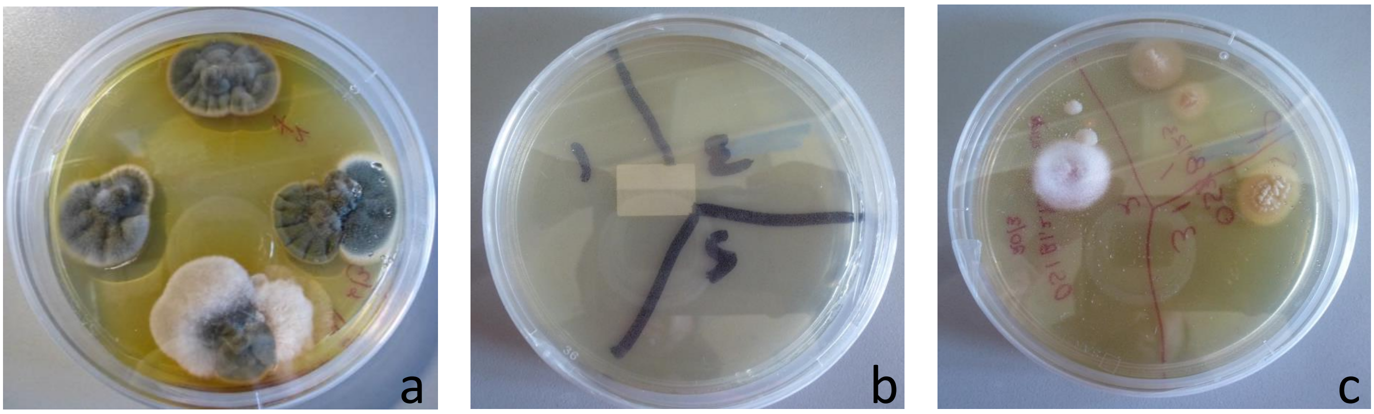

Figure S1. Isolation of fungal root endophytes. **a)** Endophytic fungi emerging from cultured roots, **b)** Clean short press plate, and **c)** Contaminated short press plate.

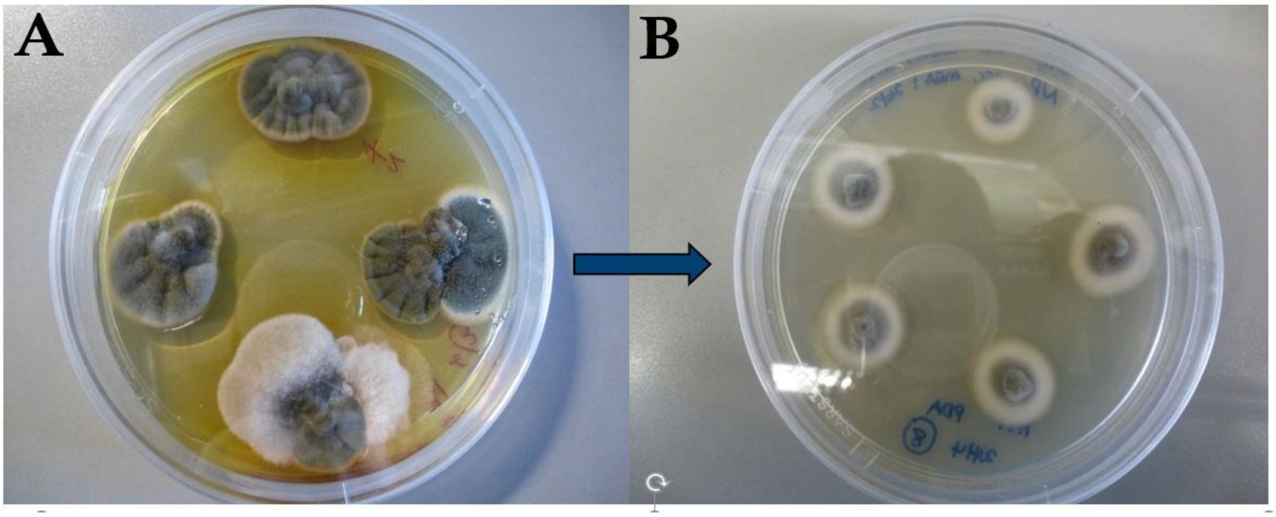

Figure S2. Isolation of fungal root endophytes from solanaceous plants. A) Endophytic fungi arising from cultured roots, and B) Pure fungal colonies.

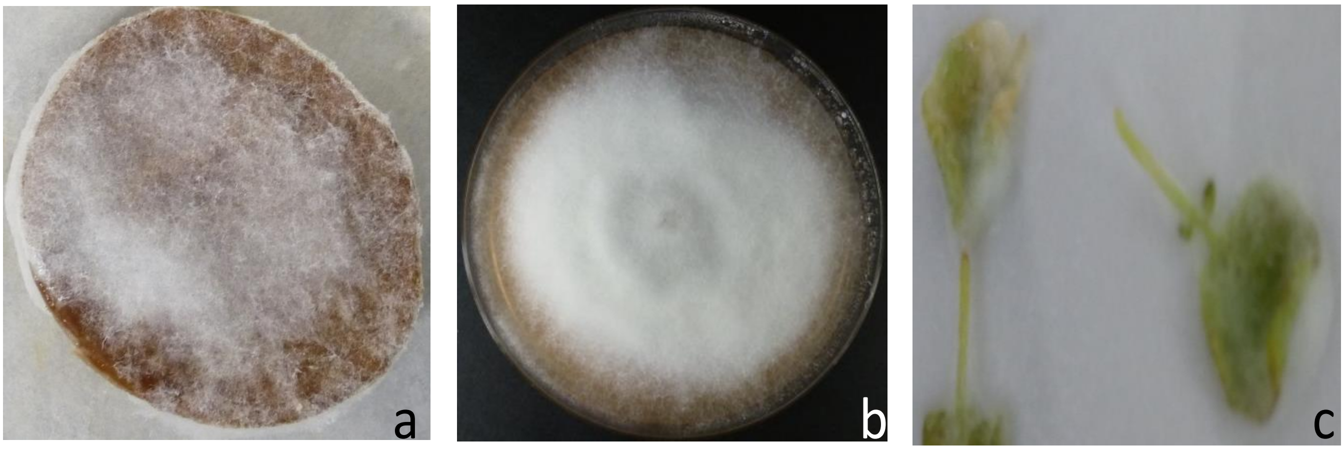

Figure S3. Isolation of *P. infestans.* **(a)** *P. infestans* mycelia growing on the surface of an inoculated potato tuber slice, **(b)** Pure colony of *P. infestans* on 20 % V8 agar, and **(c)** Mycelia of *P. infestans* on potato leaflets inoculated with the pathogen.

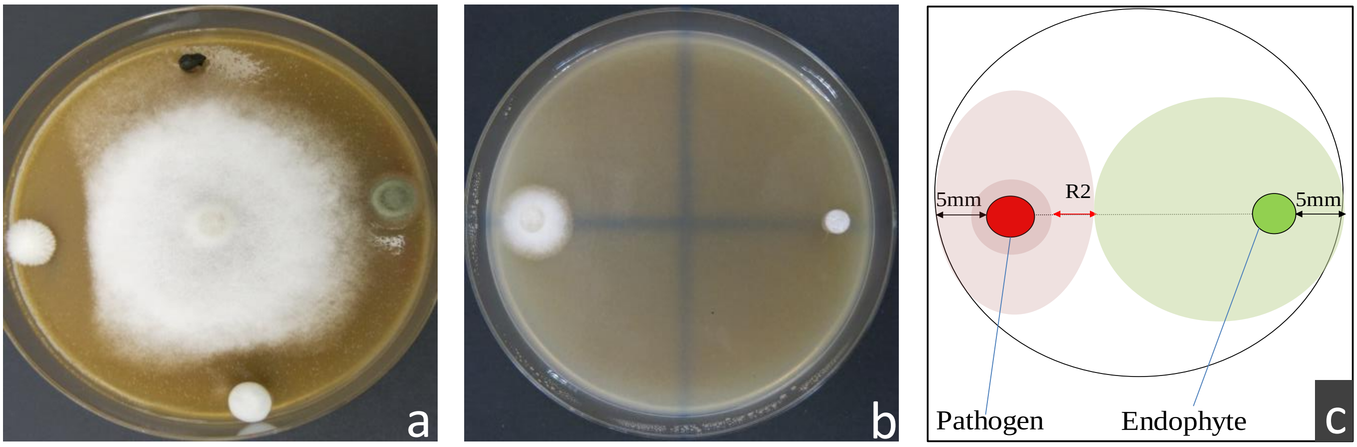

Figure S4. Dual antagonistic assay of fungal endophytes and P. infestans. (a) Co-culture of P. infestans (center colony) with four root endophytes to screen for activity against the pathogen's mycelia, (b) Setup of dual culture assay on the introduction of an endophyte 72 h after P. infestans (left colony) inoculation and, (c) Schematic representation of the measurement (R2) used in calculating percentage of inhibition.
